# Frequencies and Predictors of Negative Effects in Routine Inpatient and Outpatient Psychotherapy: Two Observational Studies

**DOI:** 10.3389/fpsyg.2020.02144

**Published:** 2020-08-25

**Authors:** Leonie Gerke, Ann-Katrin Meyrose, Inga Ladwig, Winfried Rief, Yvonne Nestoriuc

**Affiliations:** ^1^Clinical Psychology, Helmut-Schmidt-University/University of the Federal Armed Forces, Hamburg, Germany; ^2^Department of Child and Adolescent Psychiatry, Psychotherapy, and Psychosomatics, University-Medical Center Hamburg-Eppendorf, Hamburg, Germany; ^3^Clinical Psychology and Psychotherapy, University of Marburg, Marburg, Germany; ^4^Institute of Systems Neuroscience, University-Medical Center Hamburg-Eppendorf, Hamburg, Germany

**Keywords:** side effects, therapeutic malpractice, unethical therapist behavior, treatment setting, negative treatment effects, psychotherapy

## Abstract

Negative effects of psychotherapy (NEP) include side effects, malpractice, and unethical behavior. Its setting-specific frequencies and predictors are mostly unknown. The two presented studies aim to investigate NEP and its predictors systematically across different treatment settings. In study 1, *N* = 197 patients of a German outpatient center were recruited, on average, 3.76 years after the termination of psychotherapy. In study 2, data from *N* = 118 patients of two German inpatient clinics were collected at admission (*t*_0_), discharge (*t*_1_), and 9-month follow-up (*t*_2_). All participants evaluated the negative effects of their previous out- or inpatient psychotherapy with the Inventory for the Balanced Assessment of Negative Effects in Psychotherapy and *a priori* hypothesized predictors. At least one side effect was reported by 37.3% of inpatients (*t*_2_) and 15.2% of outpatients. At least one case of malpractice and unethical behavior was reported by 28.8% of inpatients (*t*_2_) and 7.1% of outpatients. Inpatients reported significantly more side effects (*U* = 14347, *z* = 4.70, *p* < 0.001, *r* = 0.26) and malpractice and unethical behavior (*U* = 14168, *z* = 5.21, *p* < 0.001, *r* = 0.29) than outpatients. Rates of severe malpractice in the form of breaking confidentiality and physical and sexual abuse were less than 1% in both settings. Predictors of side effects were prior experience with psychotherapy and current interpersonal difficulties in the outpatient setting and higher motivation for psychotherapy (*t*_0_) in the inpatient setting. Predictors of malpractice and unethical behavior were younger age in the outpatient setting and poor therapeutic alliance, prior negative experience with malpractice and unethical behavior, and higher outcome expectations in the inpatient setting. NEP are common in both, in- and outpatient settings. Inpatients are at higher risk for the NEP than outpatients. To safeguard patients’ wellbeing, the systematic assessment and distinction of side effects and malpractice and unethical behavior should gain more attention in research and clinical practice.

## Introduction

Psychotherapy is evidence-based for a wide spectrum of psychological disorders. Yet, increasing evidence for negative effects of psychotherapy (NEP) was provided over the last decade (Crawford et al., 2016; Cuijpers et al., 2018; Gawlytta et al., 2019; Moritz et al., 2019). NEP is multifaceted, including intrapersonal changes, symptom deterioration, stigmatization, and relationship problems (Ladwig et al., 2014; Abeling et al., 2018). They can arise in different treatment stages and even after termination of psychotherapy (Linden, 2013). However, many psychotherapists pay little attention to NEP in their own clinical practice and report to be unfamiliar with methods and criteria for identifying and preventing NEP (Bystedt et al., 2014; Schermuly-Haupt et al., 2018). To enhance treatment outcome and patients’ wellbeing, the early identification of NEP and its conscious handling both in research and clinical practice is called for (Meister et al., 2016; Herzog et al., 2019). As shown by the review of Jonsson et al. (2014), only 21% of randomized controlled trials of psychological treatments reported harm on the patient level. Consequently, a bias in previously reported benefit–cost ratios can be assumed (Jonsson et al., 2014). As recently demanded by the Lancet Psychiatry Commission (Holmes et al., 2018) and the extended PRISMA guidelines (Zorzela et al., 2016), the measurement of potential NEP should be the rule rather than the exception in psychotherapy research.

The research group on Risks and Side Effects of Psychotherapy recently introduced common criteria for the identification and systematic classification of NEP (Linden et al., 2018). By their definition, adverse events summarize all negative changes that are caused by either psychotherapy or external factors like critical life events. More specifically, NEP indicate negative changes with a causal relationship to psychotherapy, including side effects (SE) as well as malpractice and unethical behavior (MUB). SE cover adverse effects caused by correctly (lege artis) conducted psychotherapeutic treatments. For instance, the temporary increase of fear during exposure might represent a foreseeable and even intended SE. In contrast, MUBs, e.g., exploiting therapist behavior or sexual abuse, are the consequences of an incorrectly performed psychotherapy. According to this definition, the frequency of adverse events is typically higher than that of NEP, and the frequency of NEP is higher than that of either SE or MUB. Due to their heterogeneous origin, significance, and implications, SE and MUB need to be differentiated precisely. Finally, the research group on Risks and Side effects of psychotherapy advised measuring the severity, short-, and long-term burden on the patient and avoidability of each NEP.

According to the systematization by Linden et al. (2018), at least one SE was reported by 38.5% of patients with a current or previous depressive episode (Peth et al., 2018; Moritz et al., 2019). In line with this online survey, at least one SE was identified for 43% of outpatient cases with mixed diagnoses, as evaluated by a professional interviewer based on case reports from cognitive-behavioral therapists (Schermuly-Haupt et al., 2018). A considerably higher frequency of at least one SE was reported online by 92.9% of patients with obsessive–compulsive disorders (Moritz et al., 2015). Addressing MUB, at least one case of malpractice was reported online by 26.7% of patients with (former) depression, whereas only 8.1% noted at least one case of unethical behavior (Peth et al., 2018; Moritz et al., 2019). An unexpectedly high rate of malpractice was reported online by 89% of patients with obsessive–compulsive disorder (Moritz et al., 2015), whereas 14% of those patients noted at least one case of unethical behavior (Peth et al., 2018; Moritz et al., 2019). Causes for these differing frequencies might include heterogeneous assessment tools with varying sensitivity, patient groups with different mental disorders, and the assessment mode. The online context might, for example, entail a self-selection bias in response. In sum, these first studies consistently revealed higher frequencies for SE than for MUB.

Knowledge about predictors of NEP is highly important to prevent occurrence and, thus, improve patients’ treatment outcomes. However, systematic empirical data on predictors and mechanisms remain sparse and largely focused on positive treatment outcomes (Jonsson et al., 2014; Parry et al., 2016). The therapeutic alliance is one major factor predicting the positive treatment outcome of psychotherapy (Flückiger et al., 2018). A positive helping alliance predicted symptomatic improvement (Vogel et al., 2006; Horvath et al., 2011) and overall positive psychotherapy outcome (*r* = 0.28) in a meta-analysis (Flückiger et al., 2018). Conversely, a poor therapeutic alliance had a detrimental effect on symptom severity in a randomized-controlled trial, including participants with acute first or second episodes of a non-affective psychosis (Goldsmith et al., 2015). First studies indicate that a poor therapeutic alliance might predict the occurrence of NEP (Ladwig et al., 2014; Rozental et al., 2016; Grüneberger et al., 2017; Abeling et al., 2018). However, longitudinal or experimental data proving the causal relationship between the therapeutic alliance and the occurrence of NEP are missing. On the patient level, a small but significant positive effect of patients’ outcome expectations on posttreatment outcome (*d* = 0.24) was reported in a meta-analysis (Constantino et al., 2011). Low pretreatment outcome expectations significantly predicted more NEP (Rheker et al., 2017). In addition to alliance and expectations, patients’ motivation for psychotherapy is considered as a key for treatment adherence and effectiveness (Ryan et al., 2011). The higher motivation for psychotherapy was associated with a higher probability of achieving remission and lower posttreatment depression severity in outpatients with major depression (Zuroff et al., 2007). Further, poor patient motivation represents a risk factor for negative treatment outcome (Mohr, 1995). Another crucial risk factor for a negative outcome of psychotherapy is interpersonal difficulties (Mohr, 1995; Borkovec et al., 2002). A negative prognostic value of interpersonal difficulties for treatment outcome was shown by a randomized controlled trial, including patients with a primary diagnosis of major depressive disorder (Quilty et al., 2013). So far, implications concerning the predictive power of the earlier presented patient and therapeutic characteristics on the occurrence of NEP are restricted. Only a few studies systematically examined the occurrence of NEP or, even specifically, SE and MUB, concerning these factors. Based on the presented findings, we hypothesize that a poor therapeutic alliance, low outcome expectations, low psychotherapy motivation, and more interpersonal difficulties predict more NEP.

Besides these variables addressing therapeutic alliance and patient characteristics, context factors like prior experiences with psychotherapy and the treatment setting might be relevant for NEP (Linden et al., 2015). Research on placebo and nocebo effects revealed that prior treatment experiences either improve or reduce treatment efficacy (Kleine-Borgmann and Bingel, 2018; Colloca and Barsky, 2020). Especially, negative prior treatment experiences can drive nocebo effects and, thus, reduce treatment outcomes (Kessner et al., 2013; Colloca and Barsky, 2020). A few studies indicated that prior experience with psychotherapy constitutes a risk factor for experiencing NEP (Ladwig et al., 2014; Grüneberger et al., 2017). However, the valence of these prior experiences with psychotherapy remained unknown. Regarding treatment setting, patients from a psychiatric hospital reported more NEP than patients from a psychosomatic hospital (Rheker et al., 2017), and inpatients reported more NEP than outpatients (Ladwig et al., 2014). However, due to the small number of studies investigating setting-specific frequencies and predictors of NEP, it remains unclear whether individual clinic-specific conditions or generalizable setting-specific features like characteristics of the patient group and/or the treatment concept caused the identified setting differences in prior studies.

In summary, two pivotal limitations arise from the reviewed research on NEP: First, NEP have often been restrictively operationalized as symptom deterioration. As a result, all NEP besides symptom level changes, including important areas of patients’ life and functioning, remain disregarded (Rozental et al., 2016). Second, some previous studies neglected the precise distinction between SE and MUB. As MUB often entail a considerable burden on patients, its accurate handling presupposes a precise identification and separation from SE to safeguard patients’ wellbeing. Therefore, the aim of the present investigation was to (a) quantify the frequencies of SE and MUB in a natural out- and inpatient setting, (b) identify its cross-sectional and longitudinal predictors, and (c) compare the frequency and predictors of SE and MUB between the out- and inpatient settings.

## Materials and Methods

Two separate studies were conducted. In the cross-sectional study 1, NEP were investigated in an outpatient clinic. Data for longitudinal study 2 were collected at two inpatient clinics.

### Participants and Treatment Characteristics

#### Study 1

Participants were patients of a cognitive-behavioral outpatient center in Germany and had received psychotherapy within the last 10 years. To be eligible for the study, patients had to meet the following criteria: (a) 18 years of age or older, (b) consent to be contacted again after completion of treatment, and (c) sufficient German language skills. Patients could participate irrespective of their diagnosis. Treatment costs were covered by patients’ health care providers.

#### Study 2

Participants were recruited from two primary mental health care hospitals in Germany. Clinic A offers cognitive-behavioral treatment and Clinic B, both cognitive-behavioral and psychodynamic psychotherapies for eating disorders, anxiety disorders, obsessive–compulsive disorders, depression, borderline personality disorders, and posttraumatic stress disorders. Eligible patients met the following criteria: (a) 18 years of age or older, (b) consent to be contacted again after completion of treatment, (c) sufficient German language skills, and (d) no current alcohol or drug addiction, psychoses, brain damage, or other severe somatic disorders requiring other treatments.

### Procedure

Patients in both studies were recruited for an “Effects of Psychotherapy” study. In study 2, data from both clinics were combined for analyses to widen the sample size and to maintain confidentiality.

#### Study 1

First, files of all patients who were treated in the outpatient clinic during the last 10 years (*n* = 1,918) were screened. Of those, 502 patients met our inclusion criteria, whereas *N* = 298 agreed to participate. Participants received the postal paper–pencil questionnaire, including self-reported measures. Additional clinical and demographic data were taken from patient records. Ethical approval was obtained from the local ethics committee.

#### Study 2

**FIGURE 1:**
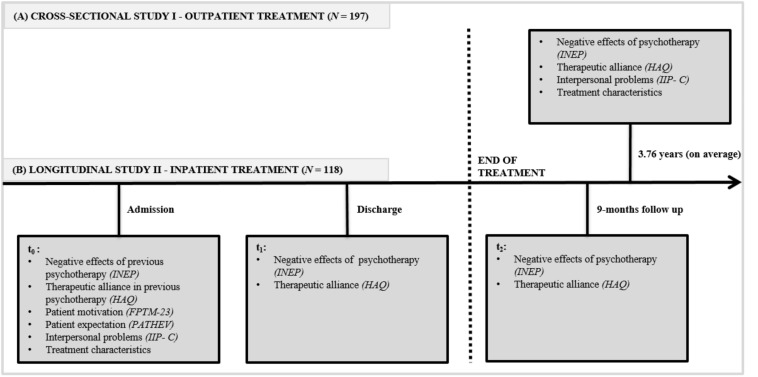
Study designs of **(A)** the cross-sectional study I and **(B)** the longitudinal study II. INEP, Inventory for the Balanced Assessment of Negative Effects of Psychotherapy; HAQ, German version of the Helping Alliance Questionnaire; IIP-C, German version of the Inventory of Interpersonal Problems; FPTM-23, German version of the Questionnaire on Psychotherapy Motivation; PATHEV, Patient questionnaire on Therapy Expectation and Evaluation; *t*_0_, at admission; *t*_1_, at discharge; *t*_2_, 9-months follow-up assessment post-treatment.

Patients were recruited during their inpatient stay by study personnel. Self-reported paper–pencil measures were administered longitudinally at admission (*t*_0_), discharge (*t*_1_), and 9-month follow-up (*t*_2_) (Figure 1). The follow-up assessment after 9 months was conducted per post. As in study 1, additional clinical and demographic data were taken from patient records. Ethical approval was obtained from the local ethics committee and the chamber of physicians of the federal state in Germany.

### Measures

#### Study 1

Sociodemographics, main diagnosis, number of diagnoses, mental health medication, previous psychotherapeutic treatments, and the number and duration of treatment sessions were gathered from patient records. Participants reported their sex, age, educational level, and marital status.

Negative effects of psychotherapy were assessed with the Inventory for the balanced Assessment of Negative Effects of Psychotherapy (INEP; Ladwig et al., 2014) via self-report, which consists of 21 items with bipolar (−1 = worse, 0 = unchanged, and 1 = better) or unipolar (0 = disagree/not applicable, 1 = agree) response options. The INEP enables the assessment of negative and positive effects of psychotherapy in seven life and functional areas, including intrapersonal changes, relationships with partner, family, friends, stigmatization and financial worries, dependency on the therapist as well as therapeutic relationship and therapeutic misconduct. Patients’ causal attributions of events to either therapy or other circumstances were assessed. We calculated the frequency of SE as the sum score of adverse events attributed to the psychotherapy (sum score of INEP items 1–15 weighted by individual attribution to treatment [“1”] or external factors [“0”], ranging from 0 to 15) in the absence of MUB (i.e., any response > “0” to items 16–21 precludes the calculation of a SE sum score). The latter exclusion criterion indicates that SE can only occur in correctly performed treatments. The frequency of reported MUB is calculated as a sum score of items 16 to 21, ranging from 0 to 6. If participants skipped certain items of the INEP (e.g., about partnership), missing values were replaced with “0,” i.e., as if no NEP was present. Items in the INEP proved reliable; Cronbach’s α = 0.86 (Ladwig et al., 2014; Herzog et al., 2019). In this investigation, Cronbach’s α values were 0.61 in study 1 and 0.87 in study 2.

The therapeutic alliance was assessed via the German version of the Helping Alliance Questionnaire (HAQ; Bassler et al., 1995). The HAQ consists of 11 items with six response options (1 = strongly disagree to 6 = strongly agree). For data analysis, the overall sum score, ranging from 11 to 66, was used with higher scores indicating a stronger therapeutic alliance. The internal consistency and validity of the HAQ are satisfactory (Bassler et al., 1995). In this investigation, Cronbach’s α values were 0.95 in study 1 and 0.96 in study 2.

Interpersonal difficulties were assessed using the German version of the self-report Inventory of Interpersonal Problems—Circumplex (Alden et al., 1990; Horowitz et al., 2000). The 64 items were offered with four response options (0 = not at all to 4 = extremely). The overall score is calculated by the mean of the sum scores of all eight subscales, ranging from 0 to 32. Higher scores indicate a higher degree of interpersonal difficulties. The Inventory of Interpersonal Problems—Circumplex’s psychometric utility is well established (Horowitz et al., 2000). In this investigation, Cronbach’s α values were 0.95 in study 1 and 0.93 in study 2. Applied instruments are displayed in Figure 1.

#### Study 2

Information regarding previous psychotherapies, inpatient treatment, mental health medication, and sociodemographic data (age, sex, educational level, and marital status) was gathered from patient records.

Positive effects, NEP, therapeutic alliance, and interpersonal difficulties were assessed as described in study 1. To increase sensitivity, answering scales of the INEP were increased to a seven-point Likert scale for bipolar items (−3 to +3 = fully agree) and four-point Likert scales for unipolar items (0 = disagree/not applicable to 3 = fully agree). To enable comparison between studies 1 and 2, answers were recoded to the format of study 1. The INEP was used to assess negative and positive effects of the inpatient treatment at discharge, 9-month follow-up, and prior experience with NEP at admission.

The motivation for psychotherapy was assessed at admission with the abridged form of the Questionnaire on Psychotherapy Motivation (Fragebogen zur Psychotherapiemotivation—FPTM-23, Schulz et al., 1995, 2000). It contains 23 items with four response options (1 = agree to 4 = fully disagree) covering six dimensions of patients’ motivation: Psychological strain (four items, range: 4 to 16), Attention from others due to psychological symptoms (three items, 3 to 12), Hope (four items, 4 to 16), Denial about needing psychological support (four items, 4 to 16), Knowledge (four items, 4 to 16), and Initiative (four items, 4 to 16). Higher sum scores indicate higher motivation for psychotherapy, except for the subscales Denial about needing psychological support and Attention from others due to psychological symptoms. For both subscales, higher sum scores indicate lower motivation for psychotherapy. Subscales’ internal consistency (α = 0.74 to 0.80) and validity are satisfactory (Schulz et al., 2003). In this study, Cronbach’s α ranged between 0.82 and 0.86, except for the subscale Knowledge (Cronbach’s α = 0.61).

Patients’ outcome expectations and treatment suitability at admission were assessed via the Patient Questionnaire on Therapy Expectation and Evaluation (Schulte, 2005) consisting of 11 items with five response options (1 = fully agree to 5 = fully disagree). Schulte (2005) identified three subscales: Hope of Improvement (four items, range: 4 to 20), Fear of Change (three items, 3 to 15), and Suitability (four items, 4 to 20). Higher sum scores indicate higher outcome expectations, except for the subscale Fear of Change; here, higher sum scores indicate lower outcome expectations. Internal consistency (α = 0.73 to 0.89) and the subscales’ construct validity with treatment outcome are satisfactory (Schulte, 2005). In this study, Cronbach’s α ranged between 0.66 and 0.87.

Prior experiences with NEP were categorized into prior experience with SE, prior experience with MUB, experience with psychotherapy without NEP, and no prior experience with psychotherapy. Applied instruments are displayed in Figure 1.

### Data Analyses

Main data analyses proceeded in three steps: First, we examined descriptive data and frequencies of NEP. Second, we conducted correlational analyses between the overall number of SE and MUB with demographic, clinical, and treatment characteristics. Correlation coefficients were chosen according to the respective scale levels (metric–metric: Pearson, metric–nominal: point–biserial [*r*_pb_], and nominal–nominal: phi-coefficient [ϕ]). Finally, we investigated the predictive power of all two-tailed significant correlates of SE and MUB within separate linear regression models. Predictors were entered simultaneously. Three sensitivity analyses were performed to test the robustness of our results against possible flaws caused by missing data imputation, memory effects, and covariating patient variables. In section “*Discussion*”, we denominate predictors as robust, if they remain stable in all sensitivity analyses. Effect sizes are presented and evaluated according to Cohen (1988), with *r* = 0.10 for small, *r* = 0.30 for medium, and *r* = 0.50 for large effect sizes. All tests were performed two-sided with an alpha error of 0.05. Data analyses were conducted using IBM SPSS Statistics software version 25.

To account for missing data, each score was calculated, if no more than one-third of the items were missing. For regression analyses, the Expectation–Maximization algorithm (Dempster et al., 1977) was used to replace all missing values in the predictor variables (below 3.1% for each predictor).

## Results

### Participants

#### Study 1

Of 298 patients who agreed to study participation, 66% (*n* = 197) completed the questionnaire. The remaining 34% (*n* = 101) dropped out for the following reasons: did not respond (*n* = 71), wrong address (*n* = 6), “didn’t have enough psychological problems” (*n* = 1), no more sessions than the initial consultation (*n* = 4), more than one-third missing data in the tested predictors (*n* = 1), and missing clinical data in patient’s file (*n* = 18). Characteristics of the analyzed sample (*n* = 197) are given in Table 1. Participants were between 23 and 77 years old and had completed their outpatient cognitive-behavioral psychotherapy on average 45.11 months ago (*SD* = 23.94), ranging from 0 to 115 months. The average number of treatment sessions was 42.03 (*SD* = 20.12), ranging from 6 to 119 treatment sessions. Most commonly, participants had a phobic (32.0%) or an affective disorder as the principal diagnosis (31.0%), followed by somatoform (20.3%), eating (5.6%), and other disorders (5.6%). For 5.6%, no information about principal diagnosis could be gathered from patient records.

**TABLE 1:** Characteristics of the outpatient and inpatient sample.

	Outpatient sample (*N* = 197)^a^	Inpatient sample (*N* = 118)^b^	Group comparison (statistical tests)
Women, *n* (%)	126 (64.0)	76 (64.4)	χ*^2^*(1) = 0.01, *p* = 0.936
Age in years, *M (SD)*	44.11 (13.58)	48.11 (9.30)	*t*(307.38) = −3.10, *p* = 0.002
In partnership, *n* (%)	120 (69.4)	88 (74.6)	χ*^2^*(1) = 0.94, *p* = 0.334
Educational level, *n* (%)			*U* = 6749, *z* = −6.12, *p* < 0.001
No school degree	3 (1.7)	5 (4.3)	
Primary degree	81 (44.8)	91 (77.8)	
Secondary degree	97 (53.6)	21 (17.9)	
Number of diagnoses, *M (SD)*	1.51 (0.84)	1.79 (0.88)	*t*(304) = −2.81, *p* = 0.005
Intake of mental health medication, *n* (%)	86 (56.6)	70 (60.9)	χ*^2^*(1) = 0.50, *p* = 0.481
Psychotherapeutic approach			
Cognitive-behavioral, *n* (%)	197 (100)	93 (78.8)	
Psychodynamic, *n* (%)	0 (0)	25 (21.2)	
Interpersonal difficulties (IIP-C), *M (SD)*^c^	10.79 (4.25)	12.87 (3.88)	*t*(264.15) = −4.44, *p* < 0.001
Therapeutic alliance (HAQ), *M (SD)*^d^	52.83 (11.98)	53.00 (13.15)	*t*(312) = −0.12, *p* = 0.908
Previous psychotherapeutic treatment(s), *n* (%)	110 (55.8)	108 (91.5)	χ*^2^*(1) = 43.49, *p* < 0.001
Prior experience with side effects	–	28 (23.7)	
Prior experience with malpractice and unethical behavior	–	58 (49.2)	
Prior experience with psychotherapy without negative effects	–	32 (27.1)	
Psychotherapy motivation (FPTM-23)^e^			
Strain, *M (SD)*	–	6.39 (2.58)	
Attention, *M (SD)*	–	7.57 (2.42)	
Hope, *M (SD)*	–	7.55 (2.83)	
Denial, *M (SD)*	–	13.90 (2.52)	
Knowledge, *M (SD)*	–	8.01 (2.82)	
Initiative, *M (SD)*	–	8.17 (3.87)	
Outcome expectations (PATHEV)^f^			
Hope of improvement, *M (SD)*	–	9.07 (3.32)	
Fear of change, *M (SD)*	–	11.42 (2.62)	
Suitability, *M (SD)*	–	9.19 (2.63)	

*SD, standard deviation; IIP-C, German version of the Inventory of Interpersonal Problems – Circumplex; HAQ, German version of the Helping Alliance Questionnaire; FPTM-23, German version of the Questionnaire on Psychotherapy Motivation; PATHEV, Patient Questionnaire on Therapy Expectation and Therapy Evaluation. ^*a*^Missing values: In partnership = 24, Educational level = 16, Number of diagnoses = 5, Intake of mental health medication = 45, Previous psychotherapeutic treatment = 1, Therapeutic alliance (HAQ) = 1. ^*b*^Missing values: Educational level = 1, Number of diagnoses = 4, Intake of mental health medication = 3. ^*c*^Averaged sum score across subscales, ranging from 0 to 32, with higher scores indicating a higher degree of interpersonal difficulties. ^*d*^Sum score, ranging from 11 to 66, with higher scores indicating stronger therapeutic alliance. ^*e*^Sum scores, ranging from 4 to 16 (except for the subscale Attention: 3 to 12), with higher scores indicating higher strain/attention/hope/denial/knowledge/initiative. ^*f*^Sum scores, ranging from 4 to 20 (except for the subscale Fear of Change: 3 to 15), with higher scores indicating a higher level of hope of improvement/fear of change/suitability.*

#### Study 2

Of 345 patients participating at *t*_0_ and/or *t*_1_, 252 (73.04%) returned the follow-up questionnaire 9 months after discharge (*t*_2_). For the present analyses, only complete datasets (*t*_0_, *t*_1_, and *t*_2_) were used, resulting in a reduced sample size of *n* = 118. Baseline characteristics are depicted in Table 1. Inpatients were between 20 to 64 years old. The duration of inpatient treatment was, on average, 45.03 days (*SD* = 15.95), ranging from 4 to 111 days. Most commonly, participants had received an affective disorder as the principal diagnosis (34.7%), followed by phobic (14.4%), eating (9.3%), somatoform (6.8%), and other disorders (30.5%). Diagnoses of five participants (4.2%) were missing.

### Frequencies and Correlates of Adverse Events and Negative and Positive Effects of Psychotherapy

#### Study 1

The majority of outpatients (76.1%) reported positive treatment effects. The most frequent positive effect being “feeling less troubled by my past” (55.8%). Half of the patients (50.3%) reported adverse events during their outpatient psychotherapy, which they perceived as caused by external factors.

At least one SE (i.e., adverse events subjectively attributed to the psychotherapy itself) was reported by 15.2% of outpatients. “Troubles finding insurance or being anxious to apply for new insurance” was the most commonly reported SE (4.6%), followed by “experiencing more conflicts in partnership” (3.0%), “experiencing more downs since the end of my therapy” (3.0%), and “worries that my colleagues or friends could find out about my psychotherapy” (2.5%). The frequencies of all SE are displayed in Table 2. More SE were reported if outpatients reported more current interpersonal difficulties (*r* = 0.19, *p* = 0.007), prior experiences with psychotherapy existed (*r*_*pb*_ = 0.18, *p* = 0.010), and less time since the termination of psychotherapy had passed (*r* = −0.16, *p* = 0.030). All correlations of NEP and patient and clinical variables are presented in Supplementary Material S1.

**TABLE 2:** Frequency of reported negative effects in outpatient (on average, 3.76 years after psychotherapy) and inpatient sample (9 months after psychotherapy).

		Outpatient sample (*N* = 197)	Inpatient sample (*N* = 118)
		
	Side effects^a^	*n* (%)	
*Intra-personal changes*	Everybody has ups and downs. Since the end of my therapy, I have experienced more downs.	6 (3.0)	19 (16.1)
	Since the end of my therapy, I have changed for the worse.	4 (2.0)	4 (3.4)
	I am more troubled by my past.	0 (0.0)	6 (5.1)
	During treatment or since the end of my therapy, I suffered from suicidal thoughts or intentions for the first time ever.	2 (1.0)	2 (1.7)
	Trusting others has become harder.	1 (0.5)	4 (3.4)
	I feel worse.	1 (0.5)	3 (2.5)
*Partner, family, and friends*	My partner and I have experienced more conflicts.	6 (3.0)	5 (4.2)
	My partner is or has been jealous of my therapist.	4 (2.0)	5 (4.2)
	The relationship with my family has worsened.	1 (0.5)	2 (1.7)
	Relationships with my friends have worsened.	1 (0.5)	1 (0.8)
*Stigmatization and financial worries*	I have had trouble finding insurance or am worried about applying for new insurance.	9 (4.6)	6 (5.1)
	I am worried that my colleagues or friends might find out about my psychotherapy.	5 (2.5)	5 (4.2)
	I have more financial worries than before.	2 (1.0)	7 (5.9)
*Dependency on therapist*	I feel addicted to my therapist.	1 (0.5)	15 (12.7)
	I have troubles making important decisions without my therapist.	4 (2.0)	6 (5.1)
	Frequency of at least one side effect	30 (15.2)	44 (37.3)
	Sum of reported side effects, mean (*SD*)	0.24(0.74)^b^	0.76(1.38)^b^
	**Malpractice and unethical behavior by therapist^c^**		
	I was hurt by what the therapist said to me.^d^	7(3.6%)	24(20.3%)
	My therapist forced me to do things I did not want to do (e.g., confrontations, role plays).^e^	8(4.1%)	17(14.4%)
	I felt personally ridiculed by my therapist.^f^	2(1.0%)	4(3.4%)
	My therapist attacked me physically.	0(0%)	1(0.8%)
	My therapist violated confidentiality.^g^	0(0%)	1(0.8%)
	I felt sexually molested by my therapist.	0(0%)	0(0%)
	Frequency of at least one case of malpractice and unethical behavior	14 (7.1)	34 (28.8)
	Sum of reported cases of malpractice and unethical behavior, mean (*SD*)	0.09(0.33)^h^	0.40(0.73)^h^

*INEP, Inventory for the Balanced Assessment of Negative Effects of Psychotherapy. ^*a*^Only adverse events that were (a) attributed to psychotherapy by the patient and (b) occurred in treatment without patient-reported MUB were counted as side effects. ^*b*^Significant difference between out- and inpatient samples (*U* = 14347, *z* = 4.70, *p* < 0.001, *r* = 0.26). ^*c*^Further patients’ explanations were voluntarily assessed with an open response option in the inpatient setting. ^*d*^Examples of inpatients’ additional explanations: “comments regarding my physical proportions,” “feeling misunderstood.” ^*e*^Examples of inpatients’ additional explanations: “open up during group sessions,” “write a letter to my wife.” ^*f*^Examples of inpatients’ additional explanations: “derogatory manner,” “feeling not taken seriously.” ^*g*^Examples of inpatients’ additional explanations: “mentioning contents of the individual sessions within the discharge report.” ^*h*^Significant difference between out- and inpatient samples (*U* = 14168, *z* = 5.21, *p* < 0.001, *r* = 0.29).*

Overall, 7.1% of outpatients reported having experienced MUB. Most frequently, they reported “feeling forced to do things I did not want to do (e.g., confrontations, role plays)” (4.1%), “being hurt by what the therapist said to me” (3.6%), or “feeling personally ridiculed by my therapist” (1%). Nobody reported sexual or physical abuse as well as violation of confidentiality (Table 2). The frequency of reported MUB was higher if outpatients were younger (*r* = −0.14, *p* = 0.046) and perceived their therapeutic alliance as less positive at discharge (*r* = −0.17, *p* = 0.015).

#### Study 2

Most inpatients (72.9%) reported positive effects of their inpatient treatment; the most frequent positive effect was “feeling less troubled by my past” (49.2%). Adverse events during psychotherapy with an external cause were mentioned by 72.9% of inpatients.

At least one SE of inpatient treatment was reported by 37.3% of respondents. Inpatients most often reported “experiencing more downs since the end of my therapy” (16.1%) and “feeling addicted to my therapist” (12.7%). The frequencies of all SE are displayed in Table 2. More SE were reported if inpatients reported higher initiative as an indicator for higher motivation for psychotherapy at treatment beginning (*r* = 0.18, *p* = 0.049).

At least one incidence of MUB was reported by 28.8% of inpatients. The most frequently reported case was “being hurt by what the therapist said to me” (20.3%), followed by *“*feeling forced to do things I did not want to do (e.g., confrontations, role plays)” (14.4%). One patient reported his/her therapist violated confidentiality by mentioning the content of the individual sessions within the discharge report. Another patient reported having been physically attacked by his or her therapist. No cases of sexual abuse were reported. Cases of MUB were more frequently reported if inpatients reported less fear of change (*r* = −0.23, *p* = 0.013) as an indicator for higher outcome expectations and less psychological strain (*r* = −0.18, *p* = 0.047) as an indicator for lower motivation for psychotherapy at treatment beginning. Additionally, more cases of MUB were reported if inpatients had prior experiences with MUB (*r_*pb*_* = 0.25, *p* = 0.005) and perceived their therapeutic alliance as poor at discharge (*r* = −0.38, *p* < 0.001).

### Predictors of Negative Effects of Psychotherapy

#### Study 1

Interpersonal difficulties, previous psychotherapeutic experience, and the time passed since psychotherapy were significantly correlated with the frequency of SE and were, thus, included in the linear regression model to predict SE. The model reached statistical significance, *F*(3,193) = 6.540, *p* < 0.001, and explained 7.8% of the variance of SEs’ frequency. All included predictors reached statistical significance. Time since psychotherapy emerged as the strongest predictor (β = −0.177, *p* = 0.011), indicating that less SE were reported if more time had passed since outpatient psychotherapy. Additionally, prior experience with psychotherapy in comparison with none (β = 0.174, *p* = 0.014) and more current interpersonal difficulties (β = 0.167, *p* = 0.017) significantly predicted a higher frequency of reported SE. All underlying statistics are displayed in Table 3.

**TABLE 3:** Linear regression analyses for the prediction of side effects in the outpatient and inpatient psychotherapy.

Predictors	*B*	*SE(B)*	β	95% CI of B	*p*	adj. *R*^2^
**Outpatient sample (*N* = 197)**						0.078
Time since end of psychotherapy (months)	−0.006	0.002	−0.177	(−0.010, −0.001)	0.011	
Previous experience with psychotherapy^a^	0.259	0.104	0.174	(0.054, 0.465)	0.014	
Interpersonal difficulties (IIP-C)^b^	0.029	0.012	0.167	(0.005, 0.053)	0.017	
**Inpatient sample (*N* = 118)**						0.025
Initiative (FPTM-23)^c^	0.065	0.033	0.182	(0.000, 0.130)	0.049	

**N* = 197. *B*, regression coefficient; *SE(B)*, standard error; β, standardized regression coefficient; 95% CI of B, 95% confidence interval of the regression coefficient; *R*^2^, variance; adj. *R*^2^, adjusted variance. IIP-C, German version of the Inventory of Interpersonal Problems—Circumplex. ^*a*^Dichotomous variable with values 0 = no previous psychotherapy, 1 = at least one previous psychotherapy. ^*b*^Averaged sum score across subscales, ranging from 0 to 32, with higher scores indicating a higher degree of interpersonal difficulties. ^*c*^Sum score, ranging from 4 to 16, with higher scores indicating higher initiative as an indicator for higher motivation for psychotherapy.*

The perceived therapeutic alliance and outpatients’ age were significantly correlated with the frequency of MUB and were, thus, included in the linear regression model to predict MUB. The model reached statistical significance, *F*(2,194) = 5.743, *p* = 0.004, explaining 4.6% of the variance in the frequency of MUB. A poor perceived therapeutic alliance (β = −0.189, *p* = 0.007) and younger age (β = −0.161, *p* = 0.023) significantly predicted a higher number of reported MUB (Table 4).

**TABLE 4:** Linear regression analyses for the prediction of malpractice and unethical behavior in the outpatient and inpatient psychotherapy.

Predictors	*B*	*SE(B)*	β	95% CI of B	*p*	adj. *R*^2^
**Outpatient sample (*N* = 197)**						0.046
Therapeutic alliance at discharge (HAQ)^a^	−0.005	0.002	−0.189	(−0.009, −0.001)	0.007	
Age	−0.004	0.002	−0.161	(−0.007, −0.001)	0.023	
**Inpatient sample (*N* = 118)**						0.218
Therapeutic alliance at discharge (HAQ)^a^	−0.019	0.005	−0.336	(−0.028, −0.009)	<0.001	
Fear of Change (PATHEV)^b^	−0.053	0.024	−0.191	(−0.100, −0.007)	0.026	
Strain (FPTM-23)^c^	−0.028	0.024	−0.098	(−0.075, 0.019)	0.244	
Previous psychotherapeutic experience^d^						
with side effects	−0.034	0.185	−0.020	(−0.400, 0.333)	0.855	
with malpractice and unethical behavior	0.362	0.163	0.249	(0.038, 0.685)	0.029	
no previous psychotherapeutic experience	−0.047	0.248	−0.018	(−0.538, 0.443)	0.849	

**B*, regression coefficient; *SE(B)*, standard error; β, standardized regression coefficient; 95% CI of B, 95% confidence interval of the regression coefficient; *R*^2^, variance; adj. *R*^2^, adjusted variance. HAQ, German version of the Helping Alliance Questionnaire; PATHEV, Patient Questionnaire on Therapy Expectation and Therapy Evaluation; FPTM-23, German version of the Questionnaire on Psychotherapy Motivation. ^*a*^Sum score, ranging from 11 to 66, with higher scores indicating stronger therapeutic alliance. ^*b*^Sum score, ranging from 3 to 15, with higher scores indicating a higher level of fear of change. ^*c*^Sum score, ranging from 4 to 16, with higher scores indicating higher psychological strain. ^*d*^Dummy-coded variable with the reference category “Prior psychotherapeutic experience without Negative Effects of Psychotherapy.”*

To test the robustness of our results, two sensitivity analyses were conducted addressing study 1. First, a comparison of analyses with and without missing data imputation was performed. The results remained unchanged. The predictors of NEP remained significant, and the effect sizes roughly equal. Second, we checked the robustness of our results regarding possible memory effects, using a reduced sample of outpatients who had completed their psychotherapy within the last 3 years (*n* = 72). Regarding SE, the predictor’s interpersonal difficulties (β = 0.332, *p* = 0.003) and prior experience with psychotherapy (β = 0.246, *p* = 0.027) remained significant. Time passed since psychotherapy did not reach statistical significance as a predictor anymore (β = −0.206, *p* = 0.059). The model explained 19.3% of the variance in the number of reported SE. Regarding the prediction of MUB, age remained a significant predictor (β = −0.313, *p* = 0.006), whereas perceived therapeutic alliance did not reach significance anymore (β = −0.212, *p* = 0.061). As in the SE-prediction model, the amount of explained variance in MUB increased to 11.7%.

#### Study 2

Initiative as an indicator for inpatients’ motivation for psychotherapy (*t*_0_) was significantly correlated with the frequency of SE and was, thus, included in the linear regression model to predict SE at 9-month follow-up. The model was statistically significant, *F*(1,116) = 3.970, *p* = 0.049, and explained 2.5% of the variance in the reported frequency of SE. A higher level of initiative as an indicator for higher motivation for psychotherapy (*t*_0_) emerged as a significant predictor of a higher frequency of SE (β = 0.182, *p* = 0.049). All underlying statistics are displayed in Table 3.

Prior experience with psychotherapy, fear of change as an indicator for inpatients’ outcome expectations (*t*_0_), psychological strain as an indicator for inpatients’ motivation for psychotherapy (*t*_0_), as well as the perceived therapeutic alliance (*t*_1_) were significantly correlated with the frequency of MUB and were, thus, included in the linear regression model to predict MUB at 9-month follow-up. The model was statistically significant, *F*(6,111) = 6.432, *p* < 0.001, and explained 21.8% of the variance in the reported frequency of MUB. Poor therapeutic alliance at discharge emerged as the strongest predictor of a higher frequency of reported cases of MUB (β = −0.336, *p* < 0.001). Prior negative experiences with MUB in comparison with prior experience with psychotherapy without NEP significantly predicted a higher frequency of reported cases of MUB (β = 0.249, *p* = 0.029). Lastly, less fear of change significantly predicted a higher amount of reported MUB (β = −0.191, *p* = 0.026). The other included predictors did not reveal a significant effect on the frequency of reported MUB (Table 4).

### Comparison Between Outpatient and Inpatient Setting

Comparisons between demographic and clinical characteristics of the out- and inpatient sample are presented in Table 1. No differences between the out- and inpatient sample were found according to the perceived therapeutic alliance (*p* = 0.908). The inpatient sample was significantly older (*p* = 0.002), had a lower educational level (*p* < 0.001), revealed a higher number of diagnoses (*p* = 0.005), and had more prior psychotherapeutic experience (*p* < 0.001). Samples differed concerning the psychotherapeutic approach, as psychodynamic treatment was only applied in the inpatient setting. Furthermore, outpatients reported less interpersonal difficulties (*p* < 0.001).

Taken together, 23.5% of all 315 participants from both studies reported at least one SE and 15.2% at least one case of MUB. Former inpatients reported, on average, significantly more SE than outpatients (*U* = 14347, *z* = 4.70, *p* < 0.001), indicating a small effect size (*r* = 0.26). Also, the number of reported MUB was significantly higher in inpatient than that in outpatient setting (*U* = 14,168, *z* = 5.21, *p* < 0.001), indicating a small but almost medium effect size (*r* = 0.29). Underlying descriptives are presented in Table 2.

In a third sensitivity analysis, we tested whether setting-specific differences in SE and MUB remained when controlling for covariating patient variables (i.e., number of diagnoses and prior experiences with psychotherapy in comparison to none). Partial correlations between the setting and number of reported SE (*r*_*pb*_ = 0.21, *p* < 0.001) as well as the setting and number of reported MUB (*r*_*pb*_ = 0.24, *p* < 0.001) remained significant when controlling for the number of diagnoses and prior experiences with psychotherapy. Values were slightly lower but comparable with correlations between the setting and number of reported SE (*r*_*pb*_ = 0.24, *p* < 0.001) and, respectively, the setting and number of MUB (*r*_*pb*_ = 0.28, *p* < 0.001) when not adjusting for these covariates.

## Discussion

The present study aimed to investigate SE and MUB from patients’ perspective in a natural out- and inpatient settings. First and foremost, the majority of participants, i.e., about three quarters in both samples, reported positive effects of their psychotherapy. At least one adverse event with an external cause during psychotherapy was reported by 50% of outpatients and by 73% of inpatients. Side effects were reported by 15% of outpatients and by 37% of inpatients. Malpractice and unethical behavior were reported by 7% of outpatients and by 29% of inpatients. Noteworthy, higher rates of both SE and MUB were reported in the inpatient than in the outpatient setting. Robust predictors of SE were prior experience with psychotherapy in comparison with none and more current interpersonal difficulties in the outpatient setting and higher motivation for psychotherapy (*t*_0_) in the inpatient setting. Robust predictors of MUB were younger age in the outpatient setting and poor therapeutic alliance, prior negative experience with MUB, and higher outcome expectations in the inpatient setting.

Adverse events seem to be a widespread phenomenon in out- and inpatient psychotherapies. Participants in these two studies reported considerable rates of both, adverse events with an external cause on the one hand and NEP on the other hand. The reported rates of at least one SE by out- and inpatients are lower than in those in previous studies (Moritz et al., 2015, 2019; Peth et al., 2018; Schermuly-Haupt et al., 2018). Consistent with previous results, frequencies of SE were higher than for MUB in both samples. Nonetheless, rates of at least one case of MUB were considerable but lower than the rates for malpractice (89%) and unethical behavior (14%) reported by patients with obsessive–compulsive disorders (Moritz et al., 2015). This discrepancy might partly be explained by the context (online vs. paper–pencil), the specific patient population, and the usage of heterogeneous instruments to assess MUB. Our results are in line with the frequencies of 26.7% for at least one case of malpractice and 8.1% for at least one case of unethical behavior in a sample of *N* = 135 patients with (former) depression reported by Peth et al. (2018). Altogether, future research on NEP should strive for a more homogenous assessment of SE and MUB to generate reliable data on prevalences of SE and MUB.

We found higher frequencies for both SE and MUB in inpatient compared with those in outpatient psychotherapy with up to medium effect sizes. Similar results were reported in a previous online investigation (Ladwig et al., 2014). These setting-specific differences are of high clinical relevance. Patients seem exposed to specific risk for experiencing NEP depending on the respective treatment setting. Relevant patient information and consent procedures should reflect these differences to enable patients and caretakers to make informed decisions about their mental health care (Nestoriuc, 2015; Trachsel and grosse Holtforth, 2019). However, to confirm these rates, larger trials and routine clinical assessments should incorporate measures of SE and MUB with the long-term goal to establish a more balanced reporting about the benefits and costs of psychological interventions. In our analyses, setting differences remained stable when controlling for covarying patient factors, namely the number of diagnoses and prior experiences with psychotherapy of out- and inpatients. Likewise, previous research did not reveal an association between patients’ symptom severity and occurrence of NEP (Rheker et al., 2017; Schermuly-Haupt et al., 2018). Hence, probably rather general setting factors than its covarying patient characteristics cause the revealed setting differences in the number of SE and MUB. Therefore, further controlled investigations should investigate the predictive power of generalizable setting-specific features like group treatment, the burden caused by close contact to fellow patients, lack of privacy, miss of familiar environment, and an externally controlled daily structure.

Overall, our prediction models for NEP, especially for SE, were not satisfactory. We found robust effects for previous experience with psychotherapy in comparison with none and more current interpersonal difficulties predicting SE in the outpatient setting. Predictor in the inpatient setting was a higher motivation for psychotherapy at treatment beginning. However, the clinical relevance of these predictors might be questioned, given the rather small percentage of explained variance. Our finding that outpatients with prior experiences with psychotherapy reported more SE than outpatients without psychotherapeutic experience is in line with the results of prior studies (Ladwig et al., 2014; Grüneberger et al., 2017). Sensitivity analysis with the reduced sample of *n* = 72 outpatients that had completed their psychotherapy less than 3 years ago led to a considerably higher variance explanation of SE. A possible explanation for the time-sensitive relationships might be that many SE are transitory and sometimes even form an inevitable part of successful psychotherapy. Hence, some SE might retrospectively be evaluated less relevant and severe; the more time has passed since therapy. Moreover, memory biases might interfere with the validity of patients’ retrospective evaluation of SE. Therefore, further longitudinal studies should address the temporal course of SE and its frequencies and significance during and after psychotherapy.

The robust predictor of MUB was younger age in the outpatient setting. Poor therapeutic alliance, prior negative experience with MUB, and higher outcome expectations emerged as the robust predictors in the inpatient setting, with a somewhat higher model fit. Taking into account the overall weak model fit and previous inconclusive research results (Crawford et al., 2016; Abeling et al., 2018; Moritz et al., 2019), the clinical relevance of the predictor age seems minor. In contrast, poor therapeutic alliance might represent a promising predictor of MUB. This finding is in line with previous research revealing the stable predictive value of the perceived therapeutic alliance for treatment outcome across treatment approaches and patient characteristics (Johansson and Jansson, 2010; Flückiger et al., 2018). However, the generalizability of our results is limited because the perceived therapeutic alliance was assessed posttreatment in both studies. Prior negative experience with MUB in comparison with prior psychotherapeutic experience without NEP was a significant predictor of MUB in the inpatient setting. Hence, only prior negative experience with MUB rather than prior experience with psychotherapy in general might depict a crucial risk factor for MUB. This is in line with previous research on nocebo effects (Kessner et al., 2013; Kleine-Borgmann and Bingel, 2018; Colloca and Barsky, 2020), indicating that prior negative experiences with psychotherapy might drive nocebo effects, in this case specifically the development of NEP. Further controlled studies are needed (a) to identify the impact of prior experiences with NEP as a relevant nocebo mechanism in psychotherapy and (b) to differentiate the predictive value of prior experiences with SE and MUB on the occurrence of NEP in subsequent psychotherapy. Our results indicate that MUB rather than SE may drive these nocebo effects. Contrary to our expectations and previous research (Rheker et al., 2017), inpatients with higher outcome expectations reported more cases of MUB. In prior studies (Ladwig et al., 2014; Abeling et al., 2018), more NEP were reported if patients’ expectations about treatment were not met. Hence, it could be assumed that both unrealistic high or low outcome expectations might depict a crucial predictor for the occurrence of NEP and especially MUB. Altogether, our findings suggest that further relevant predictors of both SE and MUB might be missing yet.

The major strengths of the present investigation are the high external validity and the broad spectrum of investigated predictors of NEP on patients, therapeutic alliance, and context level. However, several limitations need to be considered. First, some assessed constructs and measurement points vary between the out- and inpatient samples. Inpatients reported NEP at 9-month follow-up; outpatients were questioned on average 3.76 years after completion of their psychotherapy. Second, certain baseline characteristics like prior experience with psychotherapy differed between out- and inpatients, which might account for setting differences in the reported frequency of NEP. However, sensitivity analysis revealed robust setting differences when adjusting for important covarying patient characteristics. Because 92% of patients received cognitive-behavioral psychotherapy, the generalizability regarding the psychotherapeutic approaches might be questioned. Additionally, the specific type of mental health medication and other taken medications were not addressed as possible confounding variables. Causal conclusions cannot be drawn due to the correlational nature of the investigation. Hence, additional controlled studies with homogenous measurement points and experimental manipulations of possible predictors like pretreatment outcome expectations should strengthen causal implications about setting-specific differences and predictors of NEP. Third, only complete datasets (*t*_0_, *t*_1_, and *t*_2_) were used for analyses of the longitudinal study 2, and hence, dropouts were not included in the analyses of NEP. The dropout rate is high and presumably systematic, as patients experiencing NEP, especially MUB, might more frequently discontinue their treatment. Fourth, the answering scale for the INEP was adjusted for the inpatient investigation to make the instrument more sensitive, possibly resulting in not entirely comparable frequencies of NEP. Internal consistency of the INEP in the outpatient sample was not satisfactory, possibly due to the longer period since psychotherapy and accompanied memory effects, especially regarding SE.

Our findings strengthen the need for a precise differentiation between SE and MUB in research trials. To gain a more comprehensive view of NEP, future investigations should address both patients’ and therapists’ perspectives. A clear separation of SE from MUB might also reduce psychotherapists’ reservations about addressing and dealing with NEP in routine practice. NEP often form a transient and inevitable part of a lege artis conducted psychotherapy. Yet, SE and MUB that might entail serious consequences for patients need to be identified. Clinicians need to be aware of such effects and control and prevent them as much as possible, e.g., by applying techniques that trigger the fewest NEP, as it is already common practice in drug treatment.

The present investigation provides evidence that side effects, as well as malpractice and unethical behavior, are common in both out- and inpatient psychotherapies. Compared with outpatients, inpatients seem to be at higher risk for both. Due to reported limitations, additional controlled trials with specified patient groups and treatment conditions should replicate our findings to determine the predictive power of setting-specific factors. The systematic assessment and monitoring of NEP in research and clinical practice are needed. The resulting setting-, treatment-, therapist-, and patient-specific risk profiles for NEP might contribute to identify, handle, and even prevent NEP and, thus, safeguard patients’ wellbeing.

## Data Availability Statement

The raw data supporting the conclusions of this article will be made available by the authors, without undue reservation.

## Ethics Statement

The studies involving human participants were reviewed and approved by Local Ethics Committee of the Department of Psychology, University of Marburg, Germany. The patients/participants provided their written informed consent to participate in this study.

## Author Contributions

IL, WR, and YN contributed to the conception and design of work, data collection, and critical revision of the article. LG and A-KM contributed to the data analysis and interpretation and drafting of the article. All authors contributed to the article and approved the submitted version.

## Conflict of Interest

The authors declare that the research was conducted in the absence of any commercial or financial relationships that could be construed as a potential conflict of interest.

## References

[B1] Abeling B. Müller A. Stephan M. Pollmann I. de Zwaan M. (2018). Negative effekte von psychotherapie: häufigkeit und korrelate in einer klinischen stichprobe [Negative effects of psychotherapy: prevalence and correlates in a clinical sample]. *Psychother. Psychos. Med. Psychol.* 68 428–436. 10.1055/s-0043-117604 28895614

[B2] Alden L. E. Wiggins J. S. Pincus A. L. (1990). Construction of circumplex scales for the inventory of interpersonal problems. *J. Personal. Assess.* 55 521–536. 10.1080/00223891.1990.9674088 2280321

[B3] Bassler M. Potratz B. Krauthauser H. (1995). Der “Helping Alliance Questionnaire” (HAQ) von Luborsky. Möglichkeiten zur Evaluation des therapeutischen Prozesses von stationärer Psychotherapie. *Psychotherapeut* 40 23–32.

[B4] Borkovec T. D. Newman M. G. Pincus A. L. Lytle R. (2002). A component analysis of cognitive-behavioral therapy for generalized anxiety disorder and the role of interpersonal problems. *J. Consul. Clin. Psychol.* 70 288–298. 10.1037/0022-006X.70.2.288 11952187

[B5] Bystedt S. Rozental A. Andersson G. Boettcher J. Carlbring P. (2014). Clinicians’ perspectives on negative effects of psychological treatments. *Cogn. Behav. Ther.* 43 319–331. 10.1080/16506073.2014.939593 25204370 PMC4260663

[B6] Cohen J. (1988). *Statistical Power Analysis for the Behavioral Sciences*, 2nd Edn Hillsdale, NJ: Lawrence Erlbaum.

[B7] Colloca L. Barsky A. J. (2020). Placebo and nocebo effects. *New Engl. J. Med.* 382 554–561. 10.1056/NEJMra1907805 32023375

[B8] Constantino M. J. Arnkoff D. B. Glass C. R. Ametrano R. M. Smith J. Z. (2011). Expectations. *J. Clin. Psychol.* 67 184–192. 10.1002/jclp.20754 21128304

[B9] Crawford M. J. Thana L. Farquharson L. Palmer L. Hancock E. Bassett P. (2016). Patient experience of negative effects of psychological treatment: results of a national survey†. *Br. J. Psychiatry* 208 260–265. 10.1192/bjp.bp.114.162628 26932486

[B10] Cuijpers P. Reijnders M. Karyotaki E. de Wit L. Ebert D. D. (2018). Negative effects of psychotherapies for adult depression: a meta-analysis of deterioration rates. *J. Affect. Disord.* 239 138–145. 10.1016/j.jad.2018.05.050 30005327

[B11] Dempster A. P. Laird N. M. Rubin D. B. (1977). Maximum likelihood from incomplete data via the *EM* algorithm. *J. Royal Stat. Soc.* 39, 1–22. 10.1111/j.2517-6161.1977.tb01600.x

[B12] Flückiger C. Del Re A. C. Wampold B. E. Horvath A. O. (2018). The alliance in adult psychotherapy: a meta-analytic synthesis. *Psychotherapy* 55 316–340. 10.1037/pst0000172 29792475

[B13] Gawlytta R. Schwartze D. Schönherr D. Schleu A. Strauß B. (2019). Unerwünschte ereignisse durch unsachgemäß durchgeführte psychotherapie [Unwanted Events caused by incorrectly conducted psychotherapy - a pilot study of the inventory for the assessment of malpractice and its consequences in psychotherapy]. *Psychiatr. Praxis* 46 460–467. 10.1055/a-1026-1577 31683336

[B14] Goldsmith L. P. Lewis S. W. Dunn G. Bentall R. P. (2015). Psychological treatments for early psychosis can be beneficial or harmful, depending on the therapeutic alliance: an instrumental variable analysis. *Psychol. Med.* 45 2365–2373. 10.1017/S003329171500032X 25805118 PMC4501302

[B15] Grüneberger A. Einsle F. Hoyer J. Strauß B. Linden M. Härtling S. (2017). Subjektiv erlebte nebenwirkungen ambulanter verhaltenstherapie: zusammenhänge mit patientenmerkmalen, therapeutenmerkmalen und der therapiebeziehung [Subjective adverse effects during outpatient CBT: associations to patient and therapist variables and to the therapeutic alliance]. *Psychother. Psychos. Med. Psychol.* 67 338–344. 10.1055/s-0043-104930 28511238

[B16] Herzog P. Lauff S. Rief W. Brakemeier E.-L. (2019). Assessing the unwanted: a systematic review of instruments used to assess negative effects of psychotherapy. *Brain Behav.* 9:e01447. 10.1002/brb3.1447 31647202 PMC6908878

[B17] Holmes E. A. Ghaderi A. Harmer C. J. Ramchandani P. G. Cuijpers P. Morrison A. P. (2018). The lancet psychiatry commission on psychological treatments research in tomorrow’s science. *Lancet Psychiatry* 5 237–286. 10.1016/S2215-0366(17)30513-8 29482764

[B18] Horowitz L. M. Strauß B. Kordy H. (2000). *Inventar zur Erfassung Interpersonaler Probleme - Deutsche Version*, 2nd Edn Göttingen: Beltz-Test.

[B19] Horvath A. O. Del Re A. C. Flückiger C. Symonds D. (2011). Alliance in individual psychotherapy. *Psychotherapy* 48, 9–16. 10.1037/a0022186 21401269

[B20] Johansson H. Jansson J.-A. (2010). Therapeutic alliance and outcome in routine psychiatric out-patient treatment: patient factors and outcome. *Psychol. Psychother.* 83 193–206. 10.1348/147608309X472081 19793413

[B21] Jonsson U. Alaie I. Parling T. Arnberg F. K. (2014). Reporting of harms in randomized controlled trials of psychological interventions for mental and behavioral disorders: a review of current practice. *Contemp. Clin. Trials* 38 1–8. 10.1016/j.cct.2014.02.005 24607768

[B22] Kessner S. Wiech K. Forkmann K. Ploner M. Bingel U. (2013). The effect of treatment history on therapeutic outcome: an experimental approach. *JAMA Int. Med.* 173 1468–1469. 10.1001/jamainternmed.2013.6705 23780284

[B23] Kleine-Borgmann J. Bingel U. (2018). Nocebo effects: neurobiological mechanisms and strategies for prevention and optimizing treatment. *Int. Rev. Neurobiol.* 138 271–283. 10.1016/bs.irn.2018.02.005 29681330

[B24] Ladwig I. Rief W. Nestoriuc Y. (2014). Welche risiken und nebenwirkungen hat psychotherapie? - Entwicklung des inventars zur erfassung negativer effekte von psychotherapie (INEP). *Verhaltenstherapie* 24 252–263. 10.1159/000367928

[B25] Linden M. (2013). How to define, find and classify side effects in psychotherapy: from unwanted events to adverse treatment reactions. *Clin. Psychol. Psychothe.* 20 286–296. 10.1002/cpp.1765 22253218

[B26] Linden M. Strauß B. Scholten S. Nestoriuc Y. Brakemeier E.-L. Wasilewski J. (2018). Definition und entscheidungsschritte in der bestimmung und erfassung von nebenwirkungen von psychotherapie [Definition and decision-making in the determination and detection of side effects of psychotherapy]. *Psychother. Psychos. Med. Psychol.* 68 377–382. 10.1055/a-0619-5949 30286505

[B27] Linden M. Walter M. Fritz K. Muschalla B. (2015). Unerwünschte therapiewirkungen bei verhaltenstherapeutischer gruppentherapie [Undesired treatment effects in behavior group therapy: frequency and spectrum]. *Der Nervenarzt* 86 1371–1382. 10.1007/s00115-015-4297-6 25972057

[B28] Meister R. von Wolff A. Mohr H. Nestoriuc Y. Härter M. Hölzel L. (2016). Adverse event methods were heterogeneous and insufficiently reported in randomized trials on persistent depressive disorder. *J. Clin. Epidemiol.* 71 97–108. 10.1016/j.jclinepi.2015.10.007 26482955

[B29] Mohr D. C. (1995). Negative outcome in psychotherapy: a critical review. *Clin. Psychol.* 2 1–27. 10.1111/j.1468-2850.1995.tb00022.x

[B30] Moritz S. Fieker M. Hottenrott B. Seeralan T. Cludius B. Kolbeck K. (2015). No pain, no gain? Adverse effects of psychotherapy in obsessive–compulsive disorder and its relationship to treatment gains. *J. Obsess. Compul. Relat. Disord.* 5 61–66. 10.1016/j.jocrd.2015.02.002

[B31] Moritz S. Nestoriuc Y. Rief W. Klein J. P. Jelinek L. Peth J. (2019). It can’t hurt, right? Adverse effects of psychotherapy in patients with depression. *Eur. Archiv. Psychiatry Clin. Neurosci.* 269 577–586. 10.1007/s00406-018-0931-1 30088072

[B32] Nestoriuc Y. (2015). Risiken und Nebenwirkungen psychotherapeutischer Behandlung. *PiD Psychother. Dialog* 16 36–39. 10.1055/s-0041-105248

[B33] Parry G. D. Crawford M. J. Duggan C. (2016). Iatrogenic harm from psychological therapies—time to move on. *Br. J. Psychiatry* 208 210–212. 10.1192/bjp.bp.115.163618 26932481

[B34] Peth J. Jelinek L. Nestoriuc Y. Moritz S. (2018). Unerwünschte effekte von psychotherapie bei depressiven patienten – erste anwendung der positive and negative effects of psychotherapy scale (PANEPS) [Adverse Effects of Psychotherapy in Depressed Patients - First Application of the Positive and Negative Effects of Psychotherapy Scale (PANEPS)]. *Psychother. Psychos. Med. Psychol.* 68 391–398. 10.1055/s-0044-101952 29631298

[B35] Quilty L. C. Mainland B. J. McBride C. Bagby R. M. (2013). Interpersonal problems and impacts: further evidence for the role of interpersonal functioning in treatment outcome in major depressive disorder. *J. Affect. Disord.* 150 393–400. 10.1016/j.jad.2013.04.030 23726776

[B36] Rheker J. Beisel S. Kräling S. Rief W. (2017). Rate and predictors of negative effects of psychotherapy in psychiatric and psychosomatic inpatients. *Psychiatry Res.* 254 143–150. 10.1016/j.psychres.2017.04.042 28460285

[B37] Rozental A. Kottorp A. Boettcher J. Andersson G. Carlbring P. (2016). Negative effects of psychological treatments: an exploratory factor analysis of the negative effects questionnaire for monitoring and reporting adverse and unwanted events. *PLoS One* 11:e0157503. 10.1371/journal.pone.0157503 27331907 PMC4917117

[B38] Ryan R. M. Lynch M. F. Vansteenkiste M. Deci E. L. (2011). Motivation and autonomy in counseling, psychotherapy, and behavior change: a look at theory and practice 1ψ7. *Couns. Psychol.* 39 193–260. 10.1177/0011000009359313

[B39] Schermuly-Haupt M.-L. Linden M. Rush A. J. (2018). Unwanted events and side effects in cognitive behavior therapy. *Cogn. Ther. Res.* 42 219–229. 10.1007/s10608-018-9904-y

[B40] Schulte D. (2005). Messung der therapieerwartung und Therapieevaluation von patienten (PATHEV). *Z. Klinische Psychol. Psychother.* 34 176–187. 10.1026/1616-3443.34.3.176

[B41] Schulz H. Lang K. Koch U. Jürgensen R. Rüddel H. Nübling R. (2000). “Faktoren- und Itemanalysen zur Entwicklung einer Kurzform des Fragebogens zur Psychotherapiemotivation – FPTM-23,” in *Forschungsbericht Nr. 9 der Externen Evaluation der Psychosomatischen Fachklinik St. Franziska Stift, Bad Kreuznach)*, (Hamburg: Abteilungfür Medizinische Psychologie).

[B42] Schulz H. Lang K. Nübling R. Koch U. (2003). Psychometrische Überprüfung einer Kurzform des Fragebogens zur Psychotherapiemotivation - FPTM-23. *Diagnostica* 49 83–93. 10.1026//0012-1924.49.2.83

[B43] Schulz H. Nübling R. Rüddel H. (1995). Entwicklung einer Kurzform eines Fragebogens zur Psychotherapiemotivation. *Verhaltenstherapie* 5 89–95. 10.1159/000258901

[B44] Trachsel M. grosse Holtforth M. (2019). How to strengthen patients’ meaning response by an ethical informed consent in psychotherapy. *Front. Psychol.* 10:1747. 10.3389/fpsyg.2019.01747 31417470 PMC6684770

[B45] Vogel P. A. Hansen B. Stiles T. C. Götestam K. G. (2006). Treatment motivation, treatment expectancy, and helping alliance as predictors of outcome in cognitive behavioral treatment of OCD. *J. Behav. Ther. Exp. Psychiatry* 37, 247–255. 10.1016/j.jbtep.2005.12.001 16460667

[B46] Zorzela L. Loke Y. K. Ioannidis J. P. Golder S. Santaguida P. Altman D. G. (2016). PRISMA harms checklist: improving harms reporting in systematic reviews. *BMJ* 352:i157. 10.1136/bmj.i157 26830668

[B47] Zuroff D. C. Koestner R. Moskowitz D. S. McBride C. Marshall M. Bagby M. R. (2007). Autonomous motivation for therapy: a new common factor in brief treatments for depression. *Psychother. Res.* 17 137–147. 10.1080/10503300600919380

