# Supplementary material for: Frequencies and Predictors of Negative Effects in Routine Inpatient and Outpatient Psychotherapy: Two Observational Studies
Source: Front Psychol. 2020 Aug 25;11:2144. doi: 10.3389/fpsyg.2020.02144 (PMC7478145; doi:10.3389/fpsyg.2020.02144)
Supplement: Supplementary file 1 [file Table_1.DOCX]

Supplementary Material

# Supplementary Table

Supplement S1 - *Correlations of side effects, malpractice and unethical behavior (MUB), patient, and clinical data in outpatient and inpatient setting*

|  | *r* (*p*) – Inpatient sample/ Outpatient sample | | | | | | | | | | | | | | | | | | | | |  |
| --- | --- | --- | --- | --- | --- | --- | --- | --- | --- | --- | --- | --- | --- | --- | --- | --- | --- | --- | --- | --- | --- | --- |
| Measure | 1. | 2. | 3. | 4. | 5. | 6. | 7. | 8. | 9. | 10. | 11. | 12. | 13. | 14. | 15. | 16. | 17. | 18. | 19. | 20. | 21. | |
| 1. Gender^a^ | 1 | *.10 (.151)* | *.04 (.542)* | *-.07 (.342)* | *-.11 (.116)* | ***-.17 (.017)*** | *-.10 (.167)* | *.035 (.625)* | -.04^b^ (.580) | *-.07 (.354)* |  |  |  |  |  |  |  |  |  |  |  | |
| 2. Age | *.05 (.588)* | 1 | .02 (.805) | **-.14 (.046)** | .04 (.622) | .05 (.495) | -.10 (.175) | **.26 (<.001)** | .*14 (.058)* | .08 (.279) |  |  |  |  |  |  |  |  |  |  |  | |
| 3. Side effects (INEP) | *.03 (.786)* | -.02 (.820) | 1 | -.08 (.240) | .11 (.116) | .12 (.103) | -.13 (.069) | **.19 (.007)** | ***.18 (.010)*** | **-.16 (.030)** |  |  |  |  |  |  |  |  |  |  |  | |
| 4.. MUB (INEP) | *-.07 (.474)* | -.13 (.173) | **-.30 (.001)** | 1 | -.06 (.384) | .05 (.539) | **-.17 (.015)** | .04 (.574) | *-.02 (.815)* | .01 (.935) |  |  |  |  |  |  |  |  |  |  |  | |
| 5. Treatment duration^c^ | *.00 (.975)* | -.02 (.812) | .13 (.167) | -.08 (.378) | 1 | **.19 (.010)** | **.17 (.021)** | .12 (.091) | ***.24 (.001)*** | .00 (.992) |  |  |  |  |  |  |  |  |  |  |  | |
| 6. Number of diagnoses | *.05 (.590)* | .00 (.996) | -.02 (.863) | .09 (.348) | **.42 (<.001)** | 1 | .06 (.454) | **.18 (.011)** | *.11 (.133)* | -.01 (.915) |  |  |  |  |  |  |  |  |  |  |  | |
| 7. Therapeutic alliance (HAQ) | *.13 (.170)* | .12 (.188) | .17 (.065) | **-.38 (<.001)** | .12 (.211) | -.05 (.593) | 1 | **-.18 (.011)** | *-.04 (.557)* | -.10 (.180) |  |  |  |  |  |  |  |  |  |  |  | |
| 8. Interper-sonal diffi-culties (IIP-C) | *-.06 (.531)* | .07 (.464) | .00 (.976) | .11 (.239) | **.22 (.017)** | .18 (.055) | .07 (.434) | 1 | ***.18 (.013)*** | .028 (.704) |  |  |  |  |  |  |  |  |  |  |  | |
| 9. Prior experience with psychotherapy^d^ | *-* | *-* | *-* | *-* | *-* | *-* | *-* | *-* | 1 | *.08 (.263)* |  |  |  |  |  |  |  |  |  |  |  | |
| 10. Time since end of psychotherapy (months) | *-* | - | - | - | - | - | - | - | - | 1 |  |  |  |  |  |  |  |  |  |  |  | |
| 11. Hope (PATHEV) | *-.02 (.825)* | -.12 (.213) | -.02 (.855) | .18 (.052) | -.07 (.453) | **.19 (.047)** | **-.35 (<.001)** | .17 (.069) | - | - | 1 |  |  |  |  |  |  |  |  |  |  | |
| 12. Fear (PATHEV) | *-.04 (.673)* | .15 (.108) | -.05 (.592) | **-.23 (.013)** | .06 (.518) | -.01 (.924) | .16 (.090) | -.17 (.073) | - | - | **-.24 (.009)** | 1 |  |  |  |  |  |  |  |  |  | |
| 13. Suitability (PATHEV) | *.03 (.726)* | -.05 (.623) | -.01 (.908) | .11 (.252) | -.09 (.315) | .10 (.285) | **-.25 (.006)** | .17 (.071) | - | - | .**67 (<.001)** | **-.26 (.005)** | 1 |  |  |  |  |  |  |  |  | |
| 14. Strain (FPTM-23) | *-.05 (.585)* | .04 (.660) | -.09 (.313) | **-.18 (.047)** | -.15 (.111) | -.14 (.128) | .10 (.282) | **-.24 (.008)** | - | - | -.18 (.053) | .16 (.089) | -.03 (.786) | 1 |  |  |  |  |  |  |  | |
| 15. Attention (FPTM-23) | *.06 (.519)* | -.05 (.609) | .13 (.177) | .02 (.822) | -.02 (.875) | **.28 (.003)** | .04 (.699) | **.31 (.001)** | - | - | **.36 (<.001)** | .01 (.924) | .18 (.054) | **-.22 (.017)** | 1 |  |  |  |  |  |  | |
| 16. Hope (FPTM-23) | *.14 (.138)* | -.05 (.559) | .05 (.615) | .09 (.347) | .02 (.806) | .18 (.060) | **-.28 (.002)** | **.22 (.015)** | - | - | **.73 (<.001)** | **-.24 (.008)** | **.52 (<.001)** | **-.24 (.008)** | **.30 (.001)** | 1 |  |  |  |  |  | |
| 17. Denial (FPTM-23) | *.18 (.053)* | .08 (.423) | .07 (.426) | -.01 (.951) | -.07 (.432) | .09 (.365) | .17 (.062) | -.05 (.575) | - | - | **-.24 (.008)** | .13 (.171) | -.17 (.070) | .02 (.858) | -.07 (.440) | **-.19 (.037)** | 1 |  |  |  |  | |
| 18. Initiative (FPTM-23) | *.09 (.325)* | .02 (.827) | .18 (.049) | -.18 (.057) | .02 (.824) | .14 (.133) | -.06 (.498) | .09 (.326) | - | - | .11 (.221) | -.01 (.946) | .084 (.364) | .03 (.788) | .03 (.719) | .11 (.226) | -.02 (.851) | 1 |  |  |  | |
| 19. Knowledge (FPTM-23) | ***.38 (<.001)*** | -.01 (.961) | .04 (.650) | -.17 (.069) | -.05 (.575) | -.04 (.686) | .09 (.333) | **-.23 (.014)** | - | - | -.07 (.473) | -.08 (.415) | -.06 (.525) | .14 (.122) | .00 (.972) | -.03 (.725) | -.03 (.718) | .15 (.106) | 1 |  |  | |
| 20. Prior experience with side effects^e^ | .09^b^ (.358) | **-.20 (.026)** | -.01 (.956) | -.14 (.127) | -.16 (.085) | -.13 (.161) | .03 (.719) | .04 (.647) | - | - | .12 (.191) | -.11 (.222) | .16 (.076) | -.02 (.873) | .11 (.243) | .17 (.060) | -.04 (.660) | .05 (.607) | -.02 (.805) | 1 |  | |
| 21. Prior experience with MUB^e^ | -.06^b^ (.527) | **.23 (.011)** | -.15 (.103) | **.25 (.005)** | **.20 (.027)** | **.24 (.011)** | -.01 (.900) | **.33 (<.001)** | - | - | .12 (.202) | .12 (.197) | .11 (.243) | -.08 (.370) | .12 (.195) | .04 (.696) | .01 (.948) | -.09 (.323) | **-.21 (.024)** | **-.55^b^ (<.001)** | 1 | |
| 22. No prior psychotherapy^e^ | **.22^b^ (.018)** | .06 (.550) | .14 (.128) | -.13 (.178) | .14 (.122) | -.03 (.737) | .08 (.368) | **-.33 (<.001)** | - | - | -.14 (.12) | .01 (.924) | -.13 (.169) | .06 (.515) | -.17 (.061) | -.12 (.180) | .04 (.694) | .00 (.979) | **.52 (<.001)** | -.17^b^ (.065) | **-.30^b^ (.001)** | |

Note. Point-biserial correlations are displayed in italics. Significant correlations are marked in bold. HAQ = Helping Alliance Questionnaire, sum score with higher scores indicating stronger therapeutic alliance; IIP-C = German version of the Inventory of Interpersonal Problems – Circumplex, averaged sum score across subscales with higher scores indicating higher degree of interpersonal difficulties; INEP = Inventory for the balanced Assessment of Negative Effects of Psychotherapy, sum score with higher scores indicating higher frequency of side effects or respectively malpractice and unethical behavior; PATHEV = Patient questionnaire on Therapy Expectation and Evaluation, sum score with higher scores indicating higher hope/fear/suitability; FPTM-23 = German version of the Questionnaire on Psychotherapy Motivation, sum score with higher scores indicating higher strain/attention/hope/denial/ initiative/ knowledge.

^a^ Dichotomous variable with values 1 = female, 2 = male.

^b^  Phi-coefficient (*ϕ).*

^c^ Treatment duration in days in the inpatient sample and sessions in the outpatient sample.

^d^ Dichotomous variable with values 0 = no prior experience with psychotherapy, 1 = prior experience with psychotherapy.

^e^ Dummy-coded variable with the reference category ‘Prior psychotherapeutic experience without Negative Effects of Psychotherapy’.
